# Identifying the complexity of the holographic structures in strong field ionization

**DOI:** 10.1038/s41598-022-06768-6

**Published:** 2022-02-21

**Authors:** Abdelmalek Taoutioui, Károly Tőkési

**Affiliations:** Institute for Nuclear Research (ATOMKI), Bem tér 18/c, Debrecen, 4026 Hungary

**Keywords:** Atomic and molecular physics, Quantum physics

## Abstract

We present numerical investigations of the strong-field attosecond photoelectron holography by analyzing the holographic interference structures in the two-dimensional photoelectron momentum distribution (PMD) in hydrogen atom target induced by a strong infrared laser pulse. The PMDs are calculated by solving the full-dimensional time-dependent Schrödinger equation. The effect of the number of optical cycles on the PMD is considered and analyzed. We show how the complex interference patterns are formed from a single-cycle pulse to multi-cycle pulses. Furthermore, snapshots of the PMD during the time evolution are presented for a single-cycle pulse in order to track the formation of the so-called fish-bone like holographic structure. The spider- and fan-like holographic structures are also identified and investigated. We found that the fan-like structure could only be identified clearly for pulses with three or more optical cycles and its symmetry depends closely on the number of optical cycles. In addition, we found that the intensity and wavelength of the laser pulse affect the density of interference fringes in the holographic patterns. We show that the longer the wavelength, the more the holographic structures are confined to the polarization axis.

## Introduction

Strong-field tunnel ionization of atoms and molecules occurs in attosecond time scale where the resulting ultrafast electron dynamics is triggered by the tunneling ionization process which takes place in a fraction of one optical cycle of the driving laser pulse (few tens of attoseconds)^1–3^. Nowadays, the strong-field attosecond photoelectron holography (SFAPH) is a promising technique toward the dynamical imaging of ultrafast phenomena in atomic and molecular spatial scale such as chemical reactions. The clear treatment of the SFAPH which may recover the structural information of the ionized target is based on the deep understanding of the sub-cycle quantum interference occurring in the tunnel ionization regime and also on the phase decoding information hidden in the photoelectron momentum distribution^4–7^.

The induced ultrafast electron dynamics by a short and strong laser pulse in atoms and molecules is responsible for many highly nonlinear phenomena, like high order harmonic generation (HHG)^8–11^, high order above threshold ionization (HATI)^12^ and non-sequential double ionization (NSDI)^13^. Following the tunneling ionization, a portion of the ionized electron wave packet may return and scatter on the parent ion during the same optical cycle of the driving laser pulse leading to a such non-linear phenomena. In particular, these electrons represent a powerful tool for probing dynamic changes in molecular systems using laser-induced electron diffraction (LIED)^14–17^. In contract to the conventional electron diffraction experiments, the LIED imaging technique uses the self-imaging of the molecular target by its own re-scattered electrons liberated by strong-field interaction. Those electrons must be sufficiently energetic to induce a possible structure retrieval from the diffraction pattern.

On the other hand, the re-scattered electrons are also the backbone of the SFAPH imaging technique because they represent the probe electron beam of the target structure. This latter is based on the interference scenarios between the scattered and unscattered electrons liberated in the same optical cycle. Along this line an intensive work has been devoted to understand the sub-cycle electron dynamics from the momentum distributions of photoelectrons (holograms)^18–23^. The principle of photoelectron holography is based on the presence of two coherent electron wave packets. One of them is the reference (unscattered electrons) and the other is the signal (scattered electrons), which interfere between each other due to their phase difference forming holographic structures in the photoelectron momentum distribution (PMD).

The direct ionized electron wave packets emitted in different optical cycles can interfere between each other in time-domain and lead to the inter-cycle interference which explain the formation of the above threshold ionization (ATI) rings in strong-field ionization. This can be explained as a time double-slit experiment in case of few-cycle pulses and time-grating experiment in case of multi-cycle pulses^24–26^.

The interfering scattered and unscattered wave packets are released during the same optical cycle which means that the SFAPH is the consequence of the sub-cycle electron dynamics. In the literature, the well-known holographic patterns are the spider legs or commonly known as spiderlike, fishbone like, and fan-like structures. The spider legs pattern is interpreted as a consequence of the interference between two wave packets (trajectories) emitted in the same quarter of an optical cycle^19,27,28^. In general, the interference pattern in PMD is affected by the pulse duration, in case of a multi-cycle laser pulse. Electron wave packets are liberated from their initial bound state repeatedly in each optical cycle. Thus, the inter- and intra-cycle interference co-exist simultaneously during the laser-target interaction. Therefore, it is difficult to extract the electron scattering dynamics information from the PMD in case of multi-cycle pulses because of the ATI rings. Subsequently, the holographic patterns are clearly recognizable by considering few-cycle pulses. Currently, it is possible to generate single-cycle and two-cycle pulses in experiments^29–31^. In the very recent theoretical investigations, the fish-bone like and spider-like patterns have been reproduced in forward direction of the momentum distribution by using a near-infrared single-cycle pulse where the patterns are very sensitive to the carrier-envelope phase (CEP) of the pulse^28,32^. By varying the CEP, we can see both, fish-bone like and spider-like structures in the forward direction of the momentum distribution^32^.

In an attempt to disentangle and decode the electron dynamics behind the holographic structures, many trajectory based models were developed such as the semiclassical models, e.g., quasiclasical trajectory Monte Carlo (QCTMC)^33^ and the semiclassical two-step model (SCTS)^22,34^. The semiclassical two-step model assumes a maximal tunnel ionization rate at the instant of maximal electric field and when the electron is born in the continuum, it becomes a classical subject where its dynamics can be known by solving Newton equations of motion. The great advantage of the semiclassical models is that they provide an intuitive picture in terms of classical trajectories and by including the phase equation associated to each classical trajectory many holographic structures could be reproduced in the tunnel regime (for more details see^22,33,34^). We can also cite the strong-field approximation models in which the effect of the residual ion on the released photoelectrons is taken into account such as the Coulomb-Quantum orbit Strong-field approximation (CQSFA) by means of four quantum orbits^19,35^. We note, however that the quantum mechanical treatment of electron motion by solving numerically the time-dependent Schrödinger equation is still the more precise way to simulate such phenomena and can be trusted on all ionization regimes. On the other hand, semi-classical models can be used in the tunnel regime as a tool to give a realistic view on the post ionization dynamics to simulate the energy spectra and/or momentum distributions of photoelectrons.

The aim of this work is to present a quantum mechanical treatment of the electron dynamics by solving the full-dimensional time-dependent Schrödinger equation (TDSE) in order to explore the electron scattering processes encoded in the PMDs which are of utmost interest for the deep understanding of the SFAPH imaging technique. In addition, we have dealt with a half-cycle pulse where the obtained results show the presence of an interference pattern at the edge part of the PMD. In general, the scattering processes responsible for the holographic interference can be reinforced by the laser intensity or by the potential felt by the released electron.

Atomic units (a.u.) are used throughout this paper unless otherwise specified.

## Results

In order to understand how the holographic patterns are formed in strong-field tunnel ionization, we have considered the atomic hydrogen interacting with an ultrashort laser pulse. We investigate the effects of the number of optical cycles, the intensity and the wavelength of the considered laser pulse on the PMDs.

Let start with the investigation of the effect of the number of optical cycles and how it affects the momentum distribution patterns. To this end, we vary the number of optical cycles, N, of the laser pulse with a peak intensity fixed at $100~\mathrm{TW}/\mathrm{cm}^{2}$. We consider the atomic hydrogen interacting with a half-cycle pulse of carrier wavelength of $\lambda =800$ nm. The holographic patterns are very sensitive to the change of the CEP for few-cycle pulses where this laser parameter is used to monitor and control the electron motion responsible of the observed patterns (spider-like and fishbone like patterns)^27,28,32,36^. At first the carrier envelope phase is fixed at zero where the interference phenomena can be observed clearly even for a single-cycle pulse. The TDSE is only solved for the first half of a one-cycle pulse.

**Figure 1:**
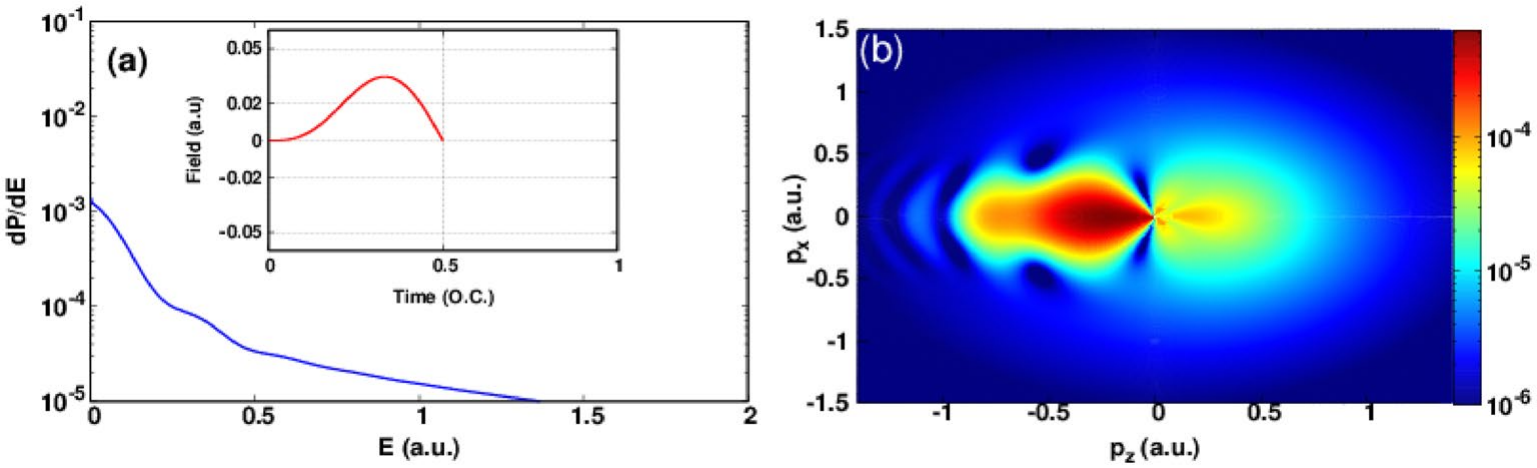
(**a**) Photoelectron spectra for a half-cycle electric field of frequency $\omega =1.55$ eV ($\lambda =800$ nm) corresponding to the intensity $1\times 10^{14}~\mathrm{W}/\mathrm{cm}^2$ ($E_0$ = 0.0534 a.u.). The carrier envelope phase is fixed at 0. Inset: electric field as a function of time in optical cycle unit. (**b**) Photoelectron momentum distribution in the plane ($p_x$,$p_z$) in logarithmic scale for the same laser pulse characteristics associated with (**a**), this heat map was generated using Gnuplot software (www.gnuplot.info), version [5.4] for Unix system.

The corresponding energy spectrum is shown in Fig. 1a. As we can see, the energy spectrum decreases exponentially as a function of photoelectron energy but we can observe the presence of a slight undulation in the energy spectrum.

**Figure 2:**
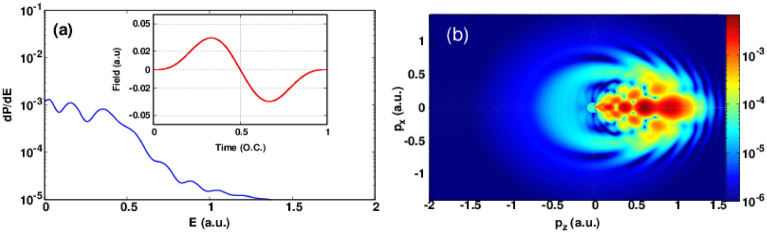
Same as Fig. 1 but the number of optical cycles N = 1.

**Figure 3:**
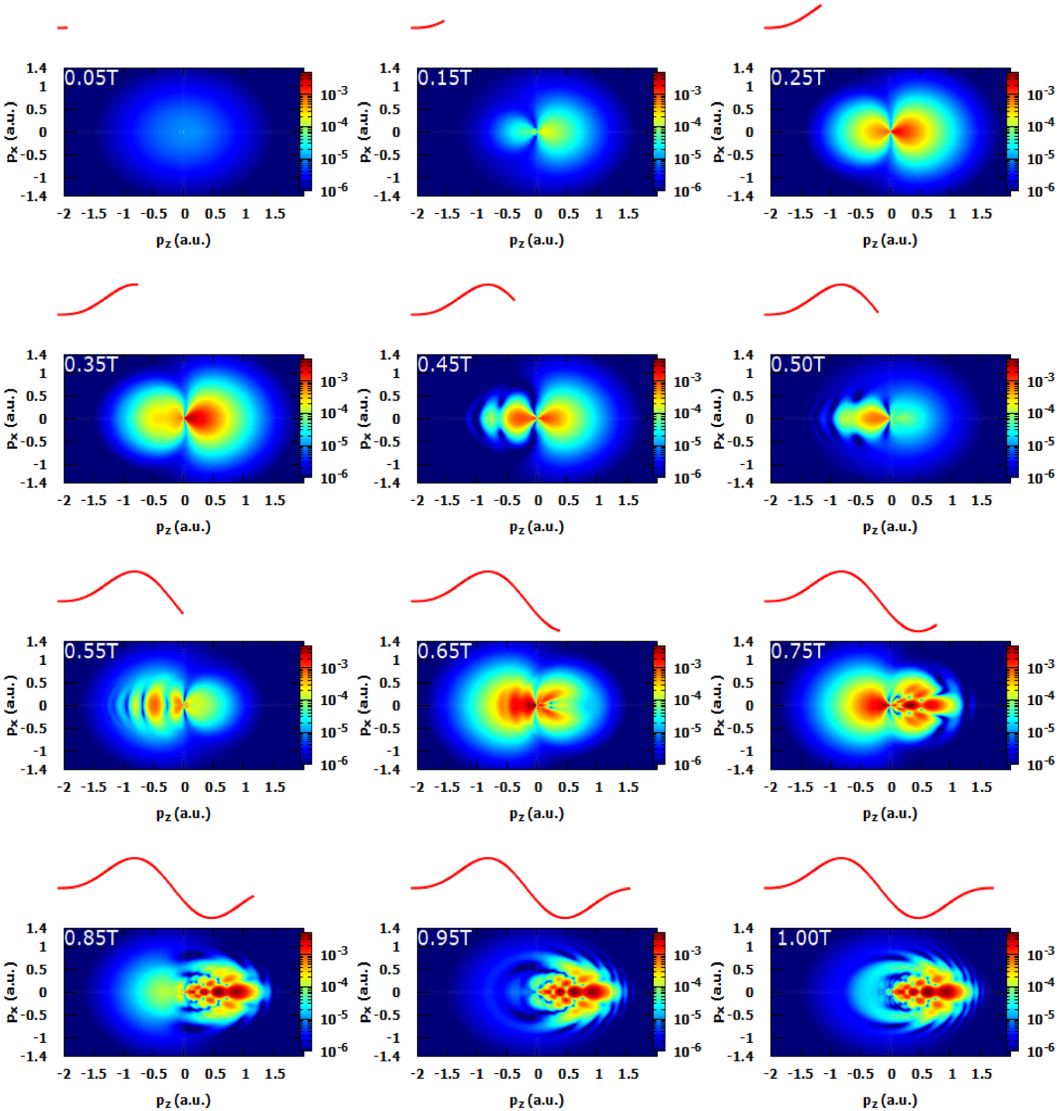
Snapshots of the photoelectron momentum distribution in case of a single-cycle pulse. The laser parameters are set as in Fig. 2. The red curve represents the electric field as a function of time. All the heat maps in this figure were generated using Gnuplot software (www.gnuplot.info), version [5.4] for Unix system.

To get a deeper insight to understand these undulations, we plot in Fig. 1b the corresponding photoelectron momentum distribution. As a first glance we observe that the most electrons are emitted in the backward direction ($p_z\le 0$) because the atomic target is predominantly tunnel ionized around the maximum of the positive electric field (see inset of Fig. 1a) whereas the emission of photoelectrons in the forward direction ($p_z\ge 0$) is less important and the existence of these photoelectrons can be justified only by the multiphoton ionization. This can be proved by the following: At the beginning of the pulse, the field is weak to produce a tunnel ionization but it is sufficient to ionize the atom via multiphoton absorption of several infrared photons. Let’s now give more attention to the pattern in the backward direction. At first we can see that we have a distorted lobe along the $p_z$ axis but we can see the formation of an interference pattern at the lobe edge. The PMD pattern in the case of one-cycle pulse at peak intensity of $1\times 10^{14}~\mathrm{W}/\mathrm{cm}^2$ is more complex than for the case of the half-cycle pulse. In Fig. 2a the photoelectron energy spectrum shows three peaks near to the ionization threshold. We found a sharp decrease around E = 0.5 a.u. and a slight undulations in higher photoelectron energies. The corresponding PMD is shown in Fig. 2b in order to analyze the peaks structure in the energy spectrum, we must note here that these peaks are not the consequence of inter-cycle (i.e temporal) interference because we are dealing here with only single-cycle pulse which confirm that the obtained interference structure is a pure sub-cycle interference or in other word holographic structure. The obtained pattern in the forward direction is known in the literature as fish-bone like structure and it is behind the three peaks discussed in the energy spectra^32^. The scenario is as follows, the released electrons in the backward direction in the first half-cycle pulse (see Fig. 1b) were driven back toward the forward direction by the negative part of the electric field and interfere with the liberated electrons in the second half-cycle leading to a fish-bone like structure. Thus, the complex electron dynamics starts in the second half-cycle of the laser pulse where the second coherent electron beam is released and interfere with the scattered one. The peaks at high photoelectron energies are responsible of the appearance of the shield structure that we observe at the edge of the obtained pattern. These latter stripes (fringes) were found in the case of a half-cycle pulse thus they are signature of interference structure between electrons released in the second half-cycle. In order to resolve the time evolution of the PMD and to have a deeper insight on how the PMD is formed at the end of the laser pulse in case of a single-cycle pulse, in Fig. 3 we plot ten snapshots of the PMD within the interaction time of *T*. The snapshots of the first-half cycle does not show any holographic structure. At the same time the snapshots taken in the second-half of the pulse show a rich interference structure holding the structural information on the ionized target. Let focus on the last four snapshots. What happens in the last quarter of single-cycle pulse ? In the snapshot at 0.75*T*, we notice the existing of holographic structure. This means that some of the released electrons in the first half-cycle encounter the new released electrons in the forward direction and interfere but a big portion of electrons is still in the backward direction and they did not scatter yet with their parent ion. In the snapshots at 0.85*T* and 0.95*T*, we can see that the fish-bone like structure has already formed. Thus, the fish-bone like structure is formed during the last quarter of the single-cycle pulse. At the end of the laser pulse (last snapshot) we notice the existence of a ring which is observable in the snapshot at 0.95T. This ring may be a signature of a backscattering of the long trajectory electrons with the parent ion later.

**Figure 4:**
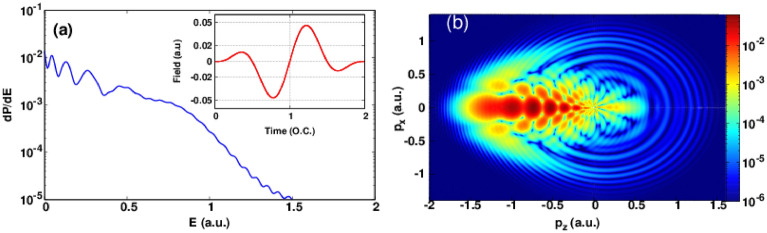
Same as Fig. 1 but for optical cycles N = 2.

Figure 4 shows the photoelectron energy (PE) spectrum with the momentum distribution for optical cycles N = 2. Fig. 4a shows also several energy peaks near to the ionization threshold but they are narrower than the case of one-cycle pulse and closer to the ionization threshold. It is important to note that the intensity felt by the target atom for one and two-cycle pulse is not the same because of the envelope function (see insets in the related figures) so the ionization yield will more important in two-cycle pulse. The associated PMD plotted in Fig. 4b shows a rich interference structure in the backward direction of the electric field where the fish-bone like structure is more clear and dense than for the case of one-cycle pulse (see Fig. 2b). In addition, the forward direction presents also a none negligible interference structure. This is because the last half-cycle of the electric field has a negative sign but it is not sufficient to drive all free electron in the forward direction. At the same time, however the low-energy photoelectrons can be forward scattered and shows a holographic structure near to the ionization threshold.

**Figure 5:**
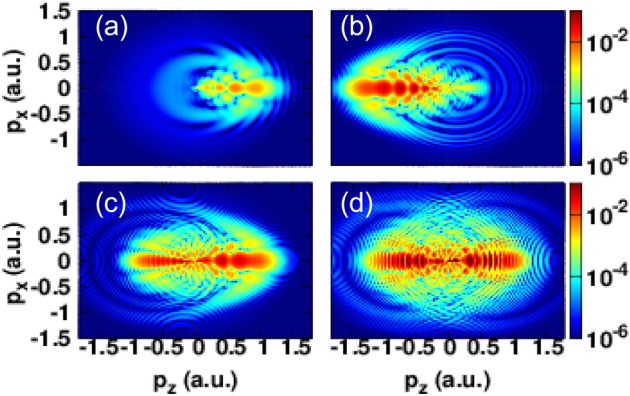
Photoelectron momentum distributions corresponding to the ionization of hydrogen induced by a strong laser pulse with different number of optical cycles N. (**a**) N = 1; (**b**) N = 2; (**c**) N = 3 ; (**d**) N = 8. The laser intensity and the carrier envelope phase are the same for all panels and are fixed respectively at $1\times 10^{14}~\mathrm{W}/\mathrm{cm}^2$ and $\varphi _{cep}=0$. All the heat maps in this figure were generated using Gnuplot software (www.gnuplot.info), version [5.4] for Unix system.

Figure 5 shows the PMDs calculated for different optical cycles namely for N = 1–3,8 at peak intensity of $1\times 10^{14}~\mathrm{W}/\mathrm{cm}^2$. The cases of one and two cycle pulses (panel (a) and (b)) we already discussed. The PMD associated to the three-cycle pulse ($N=3$) (panel (c) of Fig. 5) shows a modulated spider-like structure in the backward direction and a fish-bone like structure in the forward direction. Moreover, the fan-like structure appears near to the threshold ionization which is due to the joint influence of the electric field and the Coulomb potential on the released electrons^20^. We note that, for single cycle, two cycle and three cycle pulse the obtained PMDs are not symmetrical. The fan-like structure is clearly formed for the case of multi-cycle pulses. The holographic structure can be identified from N = 3 and for large N.

**Figure 6:**
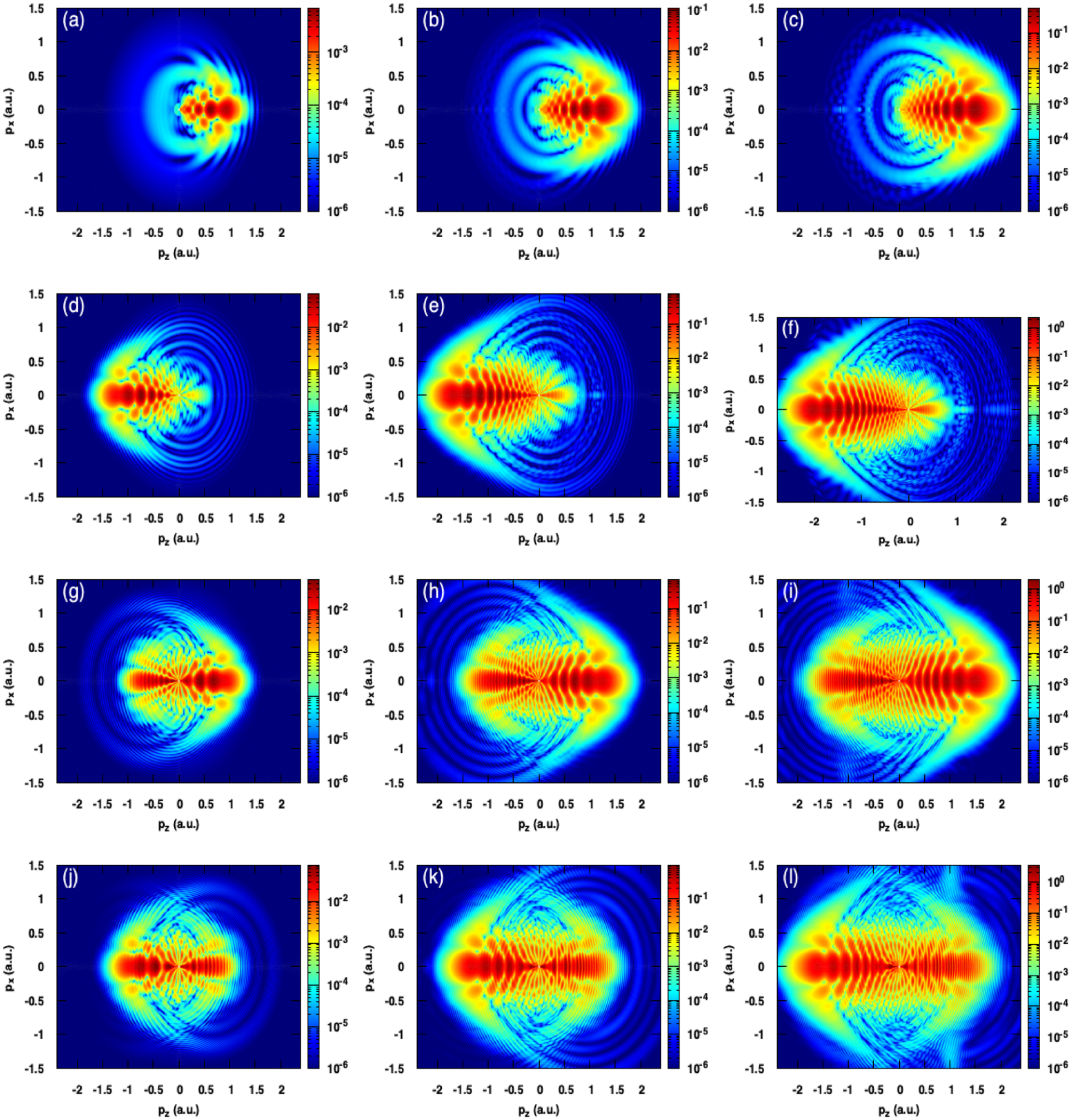
Photoelectron momentum distributions for three different peak intensities for a laser pulse with $\lambda$=800nm . Panels of the first, second and third columns correspond respectively to the peak intensities fixed at 100 TW/cm$^2$, 200 TW/cm$^2$ and 300 TW/cm$^2$. (**a**,**b**,**c**) N = 1; (**d**,**e**,**f**) N = 2; (**g**,**h**,**i**) N = 3; (**k**,**l**,**m**) N = 4. All the heat maps in this figure were generated using Gnuplot software (www.gnuplot.info), version [5.4] for Unix system.

**Figure 7:**
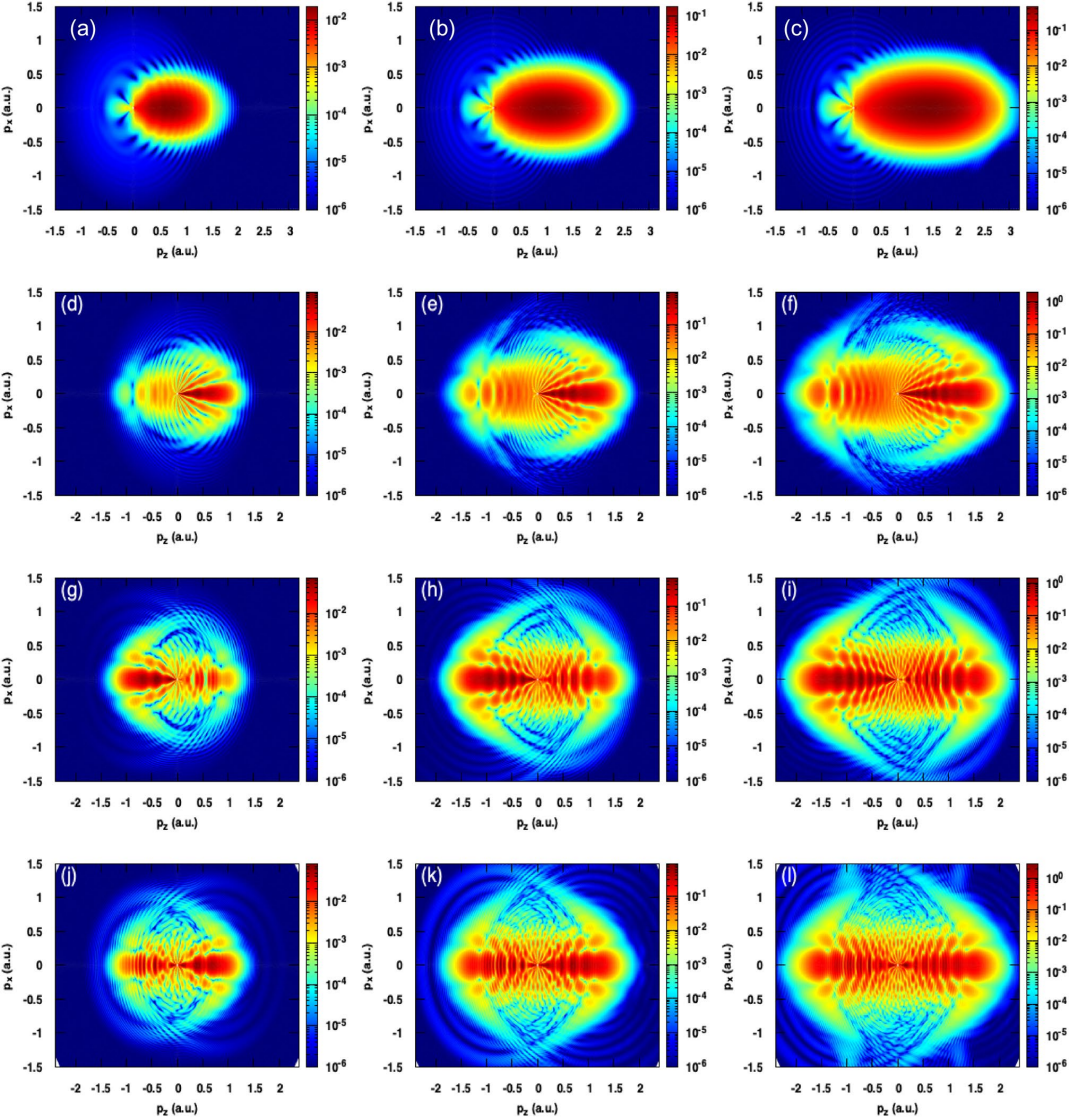
Same as Fig. 6 but for a pulse with $\varphi _{cep}=\frac{\pi }{2}$.

In Fig. 5 the panel (d) presents the PMD for an eight cycle pulse where the fan-like structure appears clearly at low photoelectron energies. In addition, the spider legs structure are also present for this multi-cycle pulse where the fish-bone like structure cannot be identified clearly due to the presence of the ATI rings. Subsequently, we can conclude that it is more relevant to use few-cycle pulses in case of holographic investigations. The formation of the fan-like pattern has two possibilities: the first can be explained by a multiphoton absorption^37^ and the second one is completely independent of the atomic structure and could be interpreted as a results of the free propagation of the released electrons in the joint presence of the residual ion and the electric field. Under this semiclassical point of view, the atomic structure does not play any contribution to the formation of momentum distribution of photoelectrons where its free propagation in the laser fields generated by the residual ion and the laser pulse is sufficient to reproduce the observed pattern^33,34^.

In order to investigate the effect of the laser intensity on the holographic patterns for the previous laser parameters, we show in Fig. 6, the PMDs for three different intensities (100 TW/cm$^2$, 200 TW/cm$^2$ and 300 TW/cm$^2$).

Increasing the number of optical cycles does not increase only the ionization yield but also changes dramatically the holographic structures where they become barely visible for multicycle pulses. On the other hand, the increase of the laser intensity does not wash out the holographic structures in the case of few-cycle pulses (N = 1–3) as we can see in the panels of the first, second and third rows of the Fig. 6. For all considered pulses N = 1–4, we can conclude that the laser intensity does not wash out the holographic patterns and increases the density of the interference fringes. Therefore, the laser intensity plays an important role in controlling the density of the interference fringes.

Figure 7 shows the PMDs as presented in Fig. 6 but with a carrier envelope phase of 90$^{\circ }$. For the case of the single cycle pulse, the electrons are emitted predominantly in the forward direction (see panels (a), (b) and (c)). This is due to the pulse shape which has a negative maximal electric field while its positive values are weak enough to tunnel ionize the target or even drive back the electrons to the backward direction. Therefore, no holographic interference is observed for N = 1. For higher values of N (N = 2–4),however ,we can observe the same conclusion as for Fig. 6 that is the intensity increases the ionization yield, extends the energy cutoff and increases the interference fringe’s density as well. Note that the patterns found for the cases of N = 2 and N = 3 are remarkably different from those obtained with $\varphi _{cep} = 0$. In particular, for N = 2, we can see the formation of the spider legs structure in the forward direction and a significant photoelectron emission in the backward direction (see panels (d), (e),(f) of Fig. 7) whereas in the case of $\varphi _{cep} = 0$ we only observed the fishbone structure (see panels (d), (e),(f) of Fig. 6). The patterns observed in the case of a pulse with N = 3 show the coexistence of well-known holographic structures in the same PMD.

**Figure 8:**
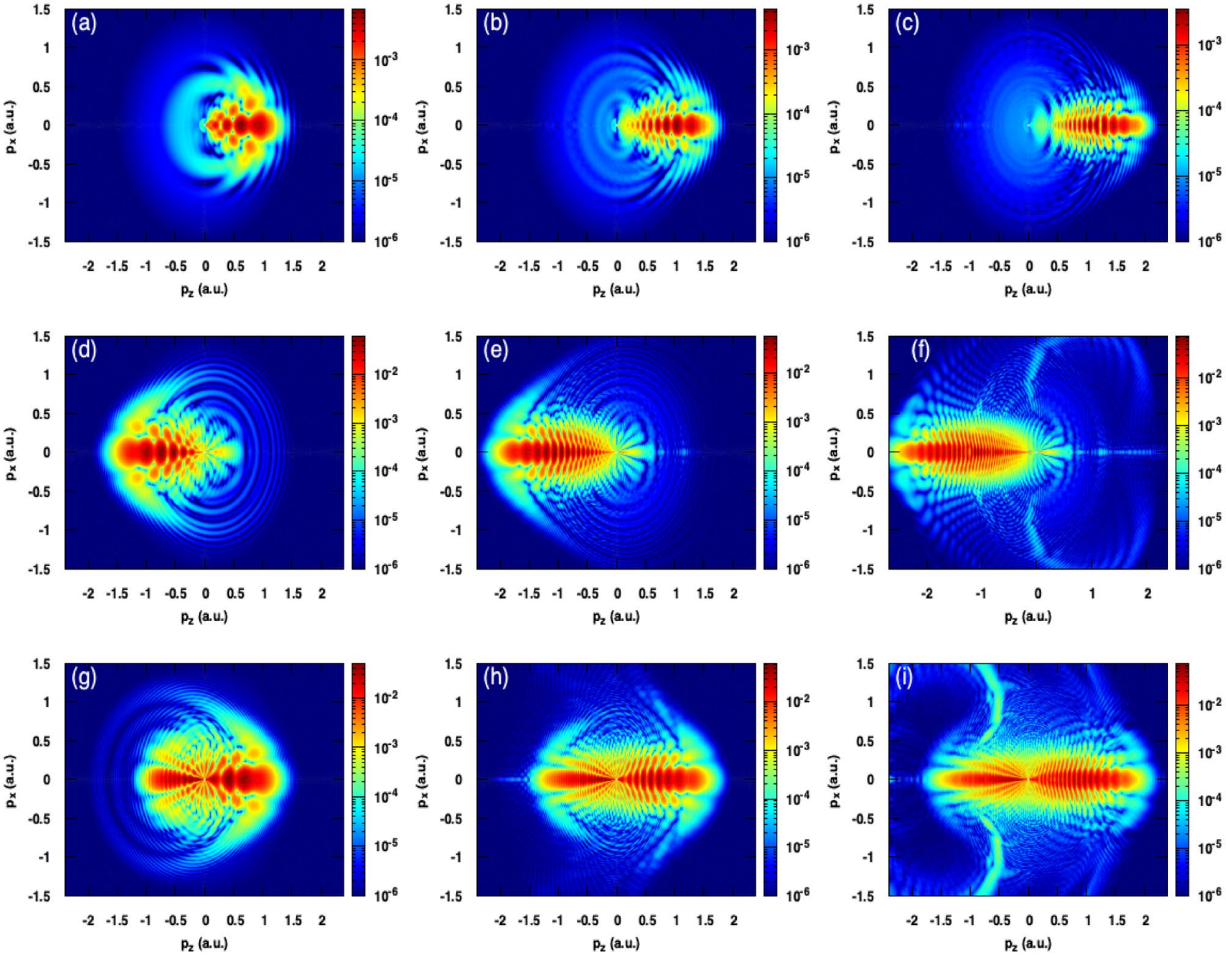
Photoelectron momentum distributions for three laser wavelengths with peak intensity and carrier envelope phase fixed respectively at 100 TW/cm$^2$ and $\varphi _{cep}$. Panels of the first, second and third column correspond respectively to the wavelengths $\lambda$= 800, 1200 and 1600 nm. (**a**,**b**,**c**) N = 1; (**d**,**e**,**f**) N = 2; (**g**,**h**,**i**) N = 3. All the heat maps in this figure were generated using Gnuplot software (www.gnuplot.info), version [5.4] for Unix system.

In order to study the effect of laser wavelength on the holographic structures, we have carried out simulations for two additional wavelenghts; $\lambda$=1200 and 1600 nm. During our simulations, the peak intensity was fixed at 100 TW/${\rm cm}^2$ and the carrier envelope phase at 0$^{\circ }$. Figure 8 shows the calculated PMDs where the first, second and third columns correspond respectively to the wavelengths 800, 1200 and 1600 nm. For all values of N, in addition to the momentum cutoff extension, we note that the longer wavelengths allow to increase the density of the interference fringes as well. Moreover, the variation of the wavelength has an interesting feature in controlling the electron emission directionality where the more wavelength is longer the more electron emission is confined to the polarization axis. It is also worth to mention that the fan-like structure becomes invisible with increasing the wavelength (see the panels (g), (h) and (i) of Fig. 8).

## Discussion

The deep understanding of the holographic patterns would allow to better model the ultrafast electron motion which represent the cornerstone of the SFAPH. This latter is considered to be a powerful tool to the dynamical imaging of the ultrafast phenomena manifesting in the atomic and molecular quantum systems where it has the high temporal and spatial resolution needed for such dynamical imaging.

In this work, we have presented numerical investigations of the strong-field attosecond photoelectron holography by analyzing the holographic interference structures in the two-dimensional photoelectron momentum distribution. As a target we used hydrogen atom and as the excitation source we used strong infrared laser pulses. The calculations was performed by solving the full-dimensional time-dependent Schrödinger equation. We have shown how the complex interference patterns are formed from a half-single-cycle pulse to a multi-cycle pulses. We have presented results demonstrating how the PMD depends on the number of optical cycles, the intensity and the wavelength of the applied laser pulse. In addition, we have also presented results taken into account two different values of the carrier envelope phase (0 and $\frac{\pi }{2}$).

It was found that the number of optical cycle is responsible of the formation of the most known holographic structures. In particular for the case of the three-cycle pulse, the PMD shows an interesting features, i.e. the spider-like, fishbone-like, shield like and fan-like holographic structures can be identified all together in the same pattern indicating that the three-cycle pulse could be a good pulse candidate to investigate the holographic structures. In this case, we can also mention that the ATI rings are less pronounced and will not wash the holographic patterns.

In case of multi-cycle pulses, in addition to the ATI rings structure, the fan-like and spider like legs can also be clearly identified but the fish-bone like structure and the shield structure can not be seen. In the recent work, we have considered a sine-squared envelope. The works taken into account the effect of the envelope function on the quantum interference phenomena by considering a trapezoidal envelope and a monochromatic field are in progress^38^. The effects of the intensity and wavelength of the laser pulse to the PMD show that they are responsible for the interference fringes density in the holographic structures. We found in particular, that the fan-like structure is very sensitive to the wavelength variation. It is interesting to note that this fan-like structure can disappear by applying longer wavelengths.

## Methods

The electron dynamics induced by a coherent laser pulse in a hydrogen atom is modeled by the time-dependent Schrödinger equation (TDSE). Here, we consider that the time-dependent interaction between a linearly polarized electric field pulse and atomic hydrogen treated in the length gauge and within the dipole approximation. Under these assumptions, the TDSE reads,

1 $$\begin{aligned} i\frac{\partial \psi (\mathbf{r },t)}{\partial t}=\Big [ -\frac{\Delta }{2} -\frac{1}{r}+ zE(t) \Big ]\psi (\mathbf{r },t), \end{aligned}$$

where the electric field is considered to be linearly polarized along the *z*-axis,

2 $$\begin{aligned} \mathbf{E}(t) = E_0sin^2\left( \frac{\pi t}{2N}\right) sin(\omega t+\phi _{cep})\mathbf {e_z}, \end{aligned}$$

with $E_0$ being the field maximum at the center of sine square envelope, N being the number of optical cycles and $\phi _{cep}$ being the carrier-envelope phase.

Since the laser is polarized linearly, the quantum magnetic number will be conserved during the interaction because of the cylindrical symmetry around the polarization axis. Therefore, in spherical coordinates the electron wave function can be expanded as

3 $$\begin{aligned} \psi (\mathbf{r },t)=\sum _{\ell =0}^{\ell _{max}} \frac{u_{\ell }(r,t)}{r}Y_{\ell }^{m=0}(\theta ). \end{aligned}$$

$\ell$ is the angular quantum number and $Y_{\ell }^{m=0}$ are the real spherical harmonics. The sum in Eq. (3) is truncated to a definite angular momentum $l_{max}$ which ensure the convergence of our simulations. By substitution of Eq. (3) in Eq. (1), we obtain the following equation

4 $$\begin{aligned} i\frac{\partial u_{\ell }(r,t)}{\partial t}=\Big [ -\frac{d^2 }{dr^2} -\frac{1}{r}+ \frac{\ell (\ell +1)}{2r^2}+ zE(t) \Big ]u_{\ell }(r,t) \end{aligned}$$

Here, the reduced radial part $u_{\ell }(r,t)$ of the wave function $\psi$ is then obtained for all $\ell$ by solving numerically the Eq. (4) using the grid method. It is based on finding the wave function on a spatial grid (box) at the end of laser pulse. All simulations presented here start from the ground state of hydrogen atom H(1s) which is generated numerically by diagonalizing the field-free Hamiltonian ($H_0=-\frac{\nabla ^2}{2}-\frac{1}{r}$). Then the solution is given for an appropriate size box from r=0 to $r_{max}=2000$. In order to avoid any undesired reflections at the box border, we have used a mask function. The kinetic energy operator (second derivative with respect to r in Eq. (4_) is approximated by three-point finite difference method and the time propagation of the wave function is carried out by using the Peaceman-Rachford scheme.

The suitable radial grid spacing and temporal step size in these simulations are $dr=0.1$ and $dt=0.02$, respectively. The convergence of our simulations is checked by performing additional calculations with smaller temporal and radial step sizes.

At the end of laser pulse, the obtained final wave function $\psi (\tau )$ contains all information about the electron dynamics. Here, we are interested in the extraction of both photoelectron spectrum and the photoelectron momentum distribution which can be analyzed to disentangle any valuable information about the ionization dynamics.

The two-dimensional photoelectron momentum distribution is obtained by projecting the final wave at the end of laser pulse on the continuum wave functions represented by the continuum waves $\psi _{\vec {k}}$. The PMD reads,

5 $$\begin{aligned} {} \frac{dP^2}{dEd\Omega } =\Big | \langle \psi _{\vec {k}} | \psi (\tau ) \rangle \Big |^2. \end{aligned}$$

E is the kinetic energy of the free electron $E=\frac{1}{2}k^2$ and the associated outgoing continuum wave for the corresponding momentum $\vec {k}$ is given by

6 $$\begin{aligned} {} \psi _{\vec {k}}^{+}(r)=\frac{4\pi }{r}\sum _{\ell =0}^{\infty }\sum _{m=-\ell }^{\ell }i^{\ell }e^{i\sigma _{\ell }(k)}\varphi _{k,\ell }(r) Y_l^m({\hat{r}})Y_{\ell }^{m*}({\hat{k}}), \end{aligned}$$

$\varphi _{k,\ell }$ being the radial part of the Coulomb wave function associated to the angular momentum $\ell$ and the kinetic energy *E* or momentum *k*. The Coulomb phase shift for a hydrogen atom is given by the analytical expression $\sigma _\ell =arg\Gamma (\ell +1+i\eta )$ where $\Gamma$ is the gamma function and $\eta =1/k$.

By substitution of Eq. (6) into Eq. (5) we find the new expression of PMD,

7 $$\begin{aligned} {} \frac{dP^2}{dkd\Omega }=\Big |\sum _{\ell =0}^{\ell _{max}}(-i)^{\ell }e^{-i\sigma _{\ell }(k)} \varphi _{k,\ell }(r)Y_{\ell }^{m*}({\hat{k}}) u_{\ell }(\tau )\Big |^2 \end{aligned}$$

The Coulomb waves $\varphi _{k,\ell }$ are numerically calculated by solving the following time-independent Schrödinger equation (TISE)

8 $$\begin{aligned} {} \Big [-\frac{1}{2}\frac{d^2}{dr^2}-\frac{1}{r}+\frac{\ell (\ell +1)}{2r^2}\Big ]\varphi _{k,l}(r)=E\varphi _{k,l}(r) \end{aligned}$$

The Eq. (8) is solved by means of a Fourth Order Runge-Kutta method and the obtained Coulomb waves are then normalized in energy by using Strömgren method^39^. Once the Coulomb waves are generated the PMD is numerically extracted using the Eq. (7).

The photoelectron spectrum can be evaluated by projection only on the radial part of the coulomb waves. Thus, we have

9 $$\begin{aligned} \frac{dP}{dE}=\int \frac{dP^2}{dEd\Omega }d\Omega \end{aligned}$$

As an alternative technique, the operator window can be also used to extract energy spectrum and momentum distribution without need to numerically generate the continuum waves^40,41^. This latter method is commonly used for calculating the energy spectra^42^.

## Data Availability

Any additional data related to this paper are available upon reasonable request to the corresponding author.
